# The role of dexamethasone in mediating the contradictory effects of Wnt antagonists SFRP2 and SFRP3 on human hair follicle growth

**DOI:** 10.1038/s41598-023-43688-5

**Published:** 2023-10-02

**Authors:** Ons Ben Hamida, Moon Kyu Kim, Mi Hee Kwack

**Affiliations:** 1 Department of Immunology, School of Medicine, Kyungpook National University, 680 Gukchaebosang-Ro, Jung-Gu, Daegu, 41944 Republic of Korea; 2 Hair Transplantation Center, Kyungpook National University Hospital, Daegu, Republic of Korea; 3 BK21 FOUR KNU Convergence Educational Program of Biomedical Sciences for Creative Future Talents, School of Medicine, Kyungpook National University, Daegu, Republic of Korea

**Keywords:** Cell biology, Molecular medicine

## Abstract

Stress can be one of the leading causes of hair loss. Stress related hormones, glucocorticoids (GCs), secretion by hair follicle have been mentioned in literature and proven to exert an inhibitory effect on hair follicle cells growth by modulating the expression of target genes related to cell proliferation and cycling. The gene modulating effect of the synthetic GC, dexamethasone (DEX), in human dermal papilla (DP) cells has been outlined in this study by mediating a contradictory effect on the expression of secreted frizzled related protein 2 (SFRP2) and SFRP3. The SFRP2 and SFRP3 possess a regulating effect on wnt signaling pathway. Their structural similarities to the cysteine-rich-domain of the frizzled receptors (FZD) allow their binding to the wnt ligands causing the blocking of the wnt ligands-receptors complex. The SFRP family members have been known as inhibitors of the wnt signaling modulating the proliferation and development of various cells. In hair follicle cells, SFRP2 activity has been reported positively on the proliferation of keratinocytes. However, the SFRP3 effect hasn’t been well addressed. Under stress, the investigation of the *mRNA* and protein expressions of SFRP members in human DP cells revealed opposite expressions where SFRP2 decreased while SFRP3 increased by DEX. The proliferation rate of hair keratinocytes outer root sheath was detected via immunofluorescence highlighting the stimulatory effect of SFRP2 and the inhibitory effect of SFRP3. Here, we sought to determine the effect of GC agonist on SFRPs expression and their effect on hair follicle growth.

## Introduction

The mammalian hair follicle is an intricately organized, complex, and dynamic organ. It is consisted of various components derived from mesenchyme, including the dermal papilla (DP) and dermal sheath (DS), as well as components originating from the epithelium, such as the outer root sheath (ORS), inner root sheath (IRS), matrix, and hair shaft. The DP enclosed by epithelial cells plays a crucial role in regulating the proliferation and cycling of hair by secreting growth factors^1,2^.

Glucocorticoids (GCs) are steroid hormones that are synthesized in the adrenal glands. These hormones play a crucial role in mediating various intracellular responses by interacting with glucocorticoid receptors (GRs), which are initially expressed in the cytoplasm of target cells. When the body experiences stress, the secretion of GCs is triggered, and these hormones then activate GRs, forming a ligand-receptor complex. This complex is able to move into the cell nucleus, where it influences gene expression and other cellular processes^3^. Apart from being synthesized in the hypothalamic–pituitary–adrenal (HPA) axis, stress-triggered hormones have also been found to be produced in hair follicles^4^. Psychological and environmental stress factors induce the release of stress-related neurohormones and stimulate the secretion of cortisol from the skin and hair follicles^5^ making GCs a peripheral equivalent of the HPA axis^6^.

Dexamethasone (DEX), a synthetic GC, promotes premature catagen stage through induction of the expression of dickkopf-1 (DKK-1), a known catagen inducer^7^. Moreover, DEX causes androgen receptors (AR) activity in a non-balding scalp^8^. For this reason, we conducted an Affymetrix chip assay on dermal papilla (DP) cells that were treated with DEX, with the aim of uncovering novel genes that are influenced by DEX treatment. Interestingly, among the secreted frizzled-related protein (SFRP) family, SFRP2 and SFRP3 showed contradictory expressions in responses to DEX. SFRP2 and SFRP3 are soluble Wnt antagonists known for their regulating effect on Wnt signaling activity. Wnts, secreted glycoproteins, possess cysteine rich domain (CRD) that allows the binding to the extracellular domain of frizzled (fzd) receptors and LDL-related protein (LRP) 5/6 co-receptors causing the mediation of hair follicle regeneration and differentiation^9,10^. The SFRP family members share structural similarities to the CRD of frizzled receptors allowing their interaction with Wnt proteins. The SFRP members sequester the binding of Wnts to their corresponding frizzled receptors by forming Wnt-SFRP complex. SFRP2 and SFRP3 have been the center of various studies discussing their inhibiting effect on the Wnt signaling pathway^11^. However, their different expressions in response to DEX had prompted us into considering a total opposite role represented by SFRP members in regulating hair growth especially that the clear function of SFRP2 and SFRP3 in the proliferation and survival of the human hair follicle cells under stress has not yet been reported.

In this study, SFRP2 and SFRP3 expressions in response to DEX treatment were compared, and their contrasting effects on human hair follicle growth were confirmed. Understanding the effects of SFRP2 and SFRP3 proteins and their mechanisms of action may reveal new therapeutic targets for treating androgenetic alopecia (AGA), a known condition for male hair loss.

## Results

### The effect of dexamethasone on the expression of SFRP2 and SFRP3 in DP cells

To investigate the effect of DEX on hair follicle, we first verified the expression of GR in human hair follicle and DP cells by performing immunofluorescence^6,8,12,13^ where it showed a significant level of GR expression in DP cells, matrix cells, and outer root sheath (ORS) cells (Supplementary Fig. 1). Next, we confirmed the isolated and cultured human hair follicles DP cells and ORS keratinocytes by performing immunostaining using respective markers. Cultured DP, known for their specific expression of α-SMA and versican markers, exhibited strong expression within DP cells, with the absence of their detection in the ORS keratinocytes. In contrast, ORS keratinocytes showed robust expression of cytokeratin 5 (CK5) and SOX9 (Supplementary Fig. 2). In DP cells, immunostaining confirmed the translocation of GR originally expressed in the cytoplasm into the nucleus after treatment with 100 nM DEX, where GR served as transcriptional factors and modulated the expression of different target genes (Supplementary Fig. 3). To test the effect of DEX on the viability of DP cells, CCK-8 assay was performed and revealed no significant inhibition or stimulation of cell growth after treatment with different doses of DEX between 1 and 500 nM (Supplementary Fig. 4).

**Figure 1:**
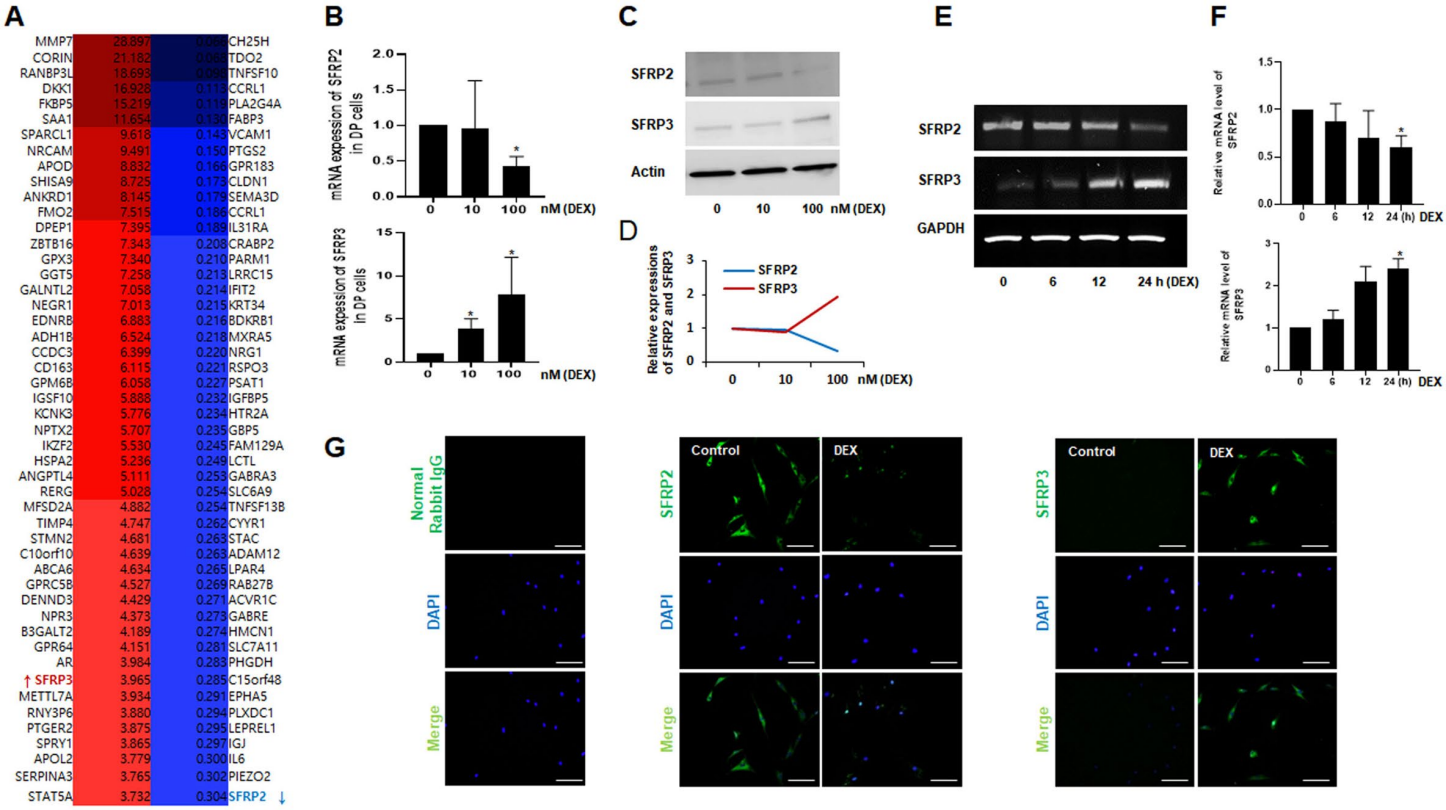
Effect of dexamethasone (DEX) on the gene and protein expression of SFRP2 and SFRP3 in human DP cells. (**A**) Representative Gene Ontology Analysis of non-balding human DP cells treated with 100 nM DEX for 24 h using Affymetrix gene chip. The up-regulated genes are shown in red**,** and the downregulated genes are shown in blue. (**B**) DP cells were incubated with increased doses of DEX (0,10,100) nM for 24 h and the mRNA expression of SFRP2 and SFRP3 were measured via qPCR. (**C**) Total protein fraction of DP cells incubated with DEX for 24 h SFRP2 and SFRP3 antibodies. Actin expression was measured to quantify and verify the integrity of protein samples. (**D**) Protein expressions of SFRP2 and SFRP3 were quantified using image J software. (**E**) DP cells were treated with 100 nM of DEX for 24 h and mRNA expressions of *SFRP2* and *SFRP3* were measured via RT-PCR. (**F**) Normalized time dependent expressions of SFRP2 and SFRP3 using image J. (**G**) Immunostaining was used to detect the protein expressions of SFRP2 and SFRP3 in DP cells in the presence or absence of 100 nM of DEX for 24 h. Normal rabbit IgG antibody was used as negative control. Bar = 0.1 mm.

Next, to identify novel genes modulated by DEX, we performed Affymetrix human gene ST. Several genes were regulated in response to 100 nM DEX (Fig. 1A and Supplementary Fig. 5). We focused on SFRP2 and SFRP3, which are known antagonists of Wnt/β-catenin signaling. Interestingly, SFRP2 (0.304-fold) was downregulated and SFRP3 (3.965-fold) upregulated by DEX (Fig. 1A).

### Expression of SFRP2 and SFRP3 by DEX in DP cells

To confirm the different expressions of SFRP2 and SFRP3 in DP cells, the corresponding *mRNA* expression was quantified by qPCR. A significant downregulation in SFRP2 expression and upregulation in SFRP3 expression were observed after treatment with DEX in a dose-dependent manner for 24 h (Fig. 1B). The protein expression of SFRP2 and SFRP3 modulated by the different doses of DEX was also verified by western blot (Fig. 1C,D and Supplementary Fig. 6). Next, we selected the highest tested dose of 100 nM of DEX to treat DP cells in a time-dependent manner (0, 6, 12, and 24 h). The *mRNA* expression of SFRP2 gradually decreased unlike the expression of SFRP3, which dramatically increased after 24 h (Fig. 1E,F). These results showed that DEX exerts the opposite effect on SFRP2 and SFRP3 expressions in a dose- and time-dependent manner. We also tested *mRNA* expression of SFRP2 and SFPR3 in ORS keratinocyte under the same conditions. No expression of these two genes was detected after DEX treatment (data not shown). We also confirmed the contradictory expression of SFRP2 and SFRP3 after treatment with 100 nM DEX for 24 h by performing immunostaining (Fig. 1G). SFRP2 expression was high in the cytoplasm of human DP cells in the absence of DEX but dramatically decreased after DEX treatment. In contrast, the weakly expressed SFRP3 in cultured DP cells has increased after DEX treatment. Normal rabbit IgG was used as negative control to ensure specific staining of the cells.

### The expression of DEX mediated SFRP2 and SFRP3 is regulated by GR

**Figure 2:**
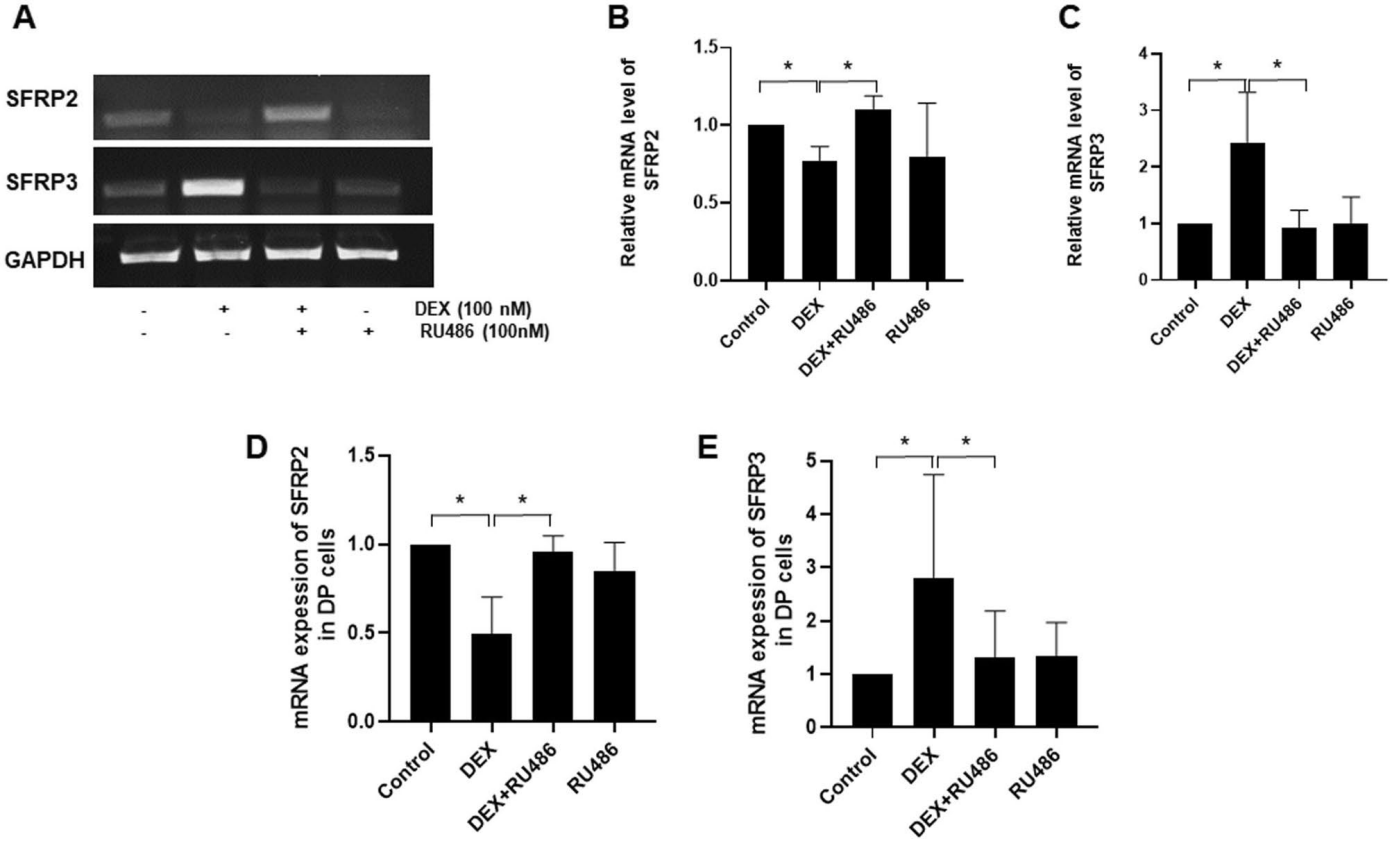
The regulatory role of GR in DEX mediated expression of SFRP2 and SFRP3 in DP cells. (**A**) DP cells were cultured with or without 100 nM of DEX, 100 nM of RU486 and a combination of 100 nM of DEX and RU486 for 24 h and then the mRNA expressions of *SFRP2* (**B**) and *SFRP3* (**C**) were quantified using RT-PCR and normalized by image J. (**D, E**) qPCR was also used for verification.

The DEX-induced decrease in SFRP2 expression was rescued after treatment of DP cells with RU486, a synthetic GR antagonist. In contrast, the expression of SFRP3 was reduced in response to RU486 treatment (Fig. 2). These results indicate the expression of SFRP2 and SFRP3 in response to DEX is regulated by GR and confirm the modulatory effect of the stress hormone DEX on the expression of SFRP2 and SFRP3 where it inhibits one whereas it induces the other. Whether the SFRP2 and SFRP3 have opposite effects by DEX on the proliferation of DP cells and their mechanisms of action on hair follicle growth is yet to be investigated.

### The effect of SFRP2 and SFRP3 on the Wnt/β-catenin signaling in human DP cells

**Figure 3:**
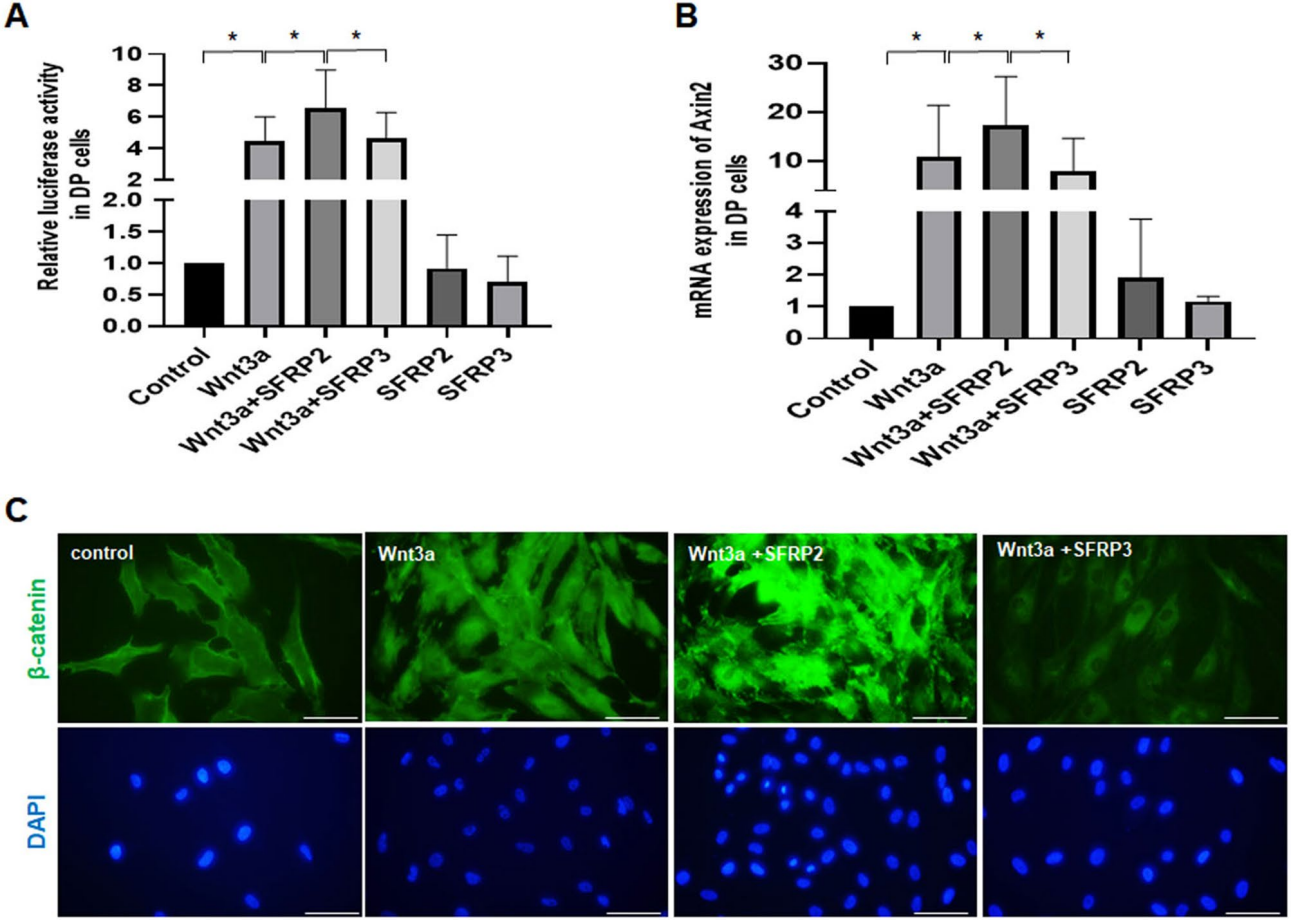
The effect of SFRP2 and SFRP3 on the Wnt/β-catenin signaling in DP cells. (**A**) DP cells were incubated with 100 ng/ml of rhSFRP2 or rhSFRP3 for 12 h in the presence or absence of 250 ng/ml or rhwnt3a and the wnt**/**β-catenin activity was measured using the luciferase reporter assay. (**B**) The mRNA expression of wnt target gene *Axin2* was also measured in DP cells using qPCR. (**C**) DP cells were incubated for 6 h in the presence or the absence of rhwnt3a, rhwnt3a combined treatment rhSFRP2 or rhwnt3a combined with rhSFRP3 and the β-catenin activation was detected via immunofluorescence. DAPI nuclear staining of the cells was also performed. Bar = 0.1 mm.

To assess the effect of SFRP2 and SFRP3 on the Wnt/β-catenin activity, we transfected with β-catenin-responsive T-cell factor reporter plasmid (pTopflash) in DP cells. Cells co-treated with recombinant human wnt3a (rhwnt3a) and rhSFRP2 for 12 h showed an increase in β-catenin activity compared to rhwnt3a treated cells only (Fig. 3A). In consistence with the previous data^14^, the combination of rhwnt3a and rhSFRP2 treatment significantly increased Wnt3a-mediated elevation of β-catenin activity in the DP cells. Unlike SFPP2, SFRP3 did not show any significant effect on Wnt3a-mediated elevation of β-catenin activity in human DP cells. In addition, the treatment of either rhSFRP2 or rhSFRP3 without Wnt3a didn’t significantly increase the β-catenin activity. Next, we quantified the *mRNA* expression of Wnt target gene Axin2 to confirm the previous findings. qPCR results showed highest *mRNA* expression of Axin2 when treated with rhwnt3a and rhSFRP2 combined compared to rhwnt3a and rhSFRP3 in DP cells (Fig. 3B). In line with the above results, treatment with rhWnt3a resulted in increment of β-catenin protein level, and rhWnt3a-induced β-catenin was augmented when rhSFRP2 treatment was combined with rhWnt3a. In contrast, when a combined treatment with Wnt3a and SFRP3 was added to the culturing milieu, the expression of β-catenin protein resembled that of the control group (Fig. 3C).

### The effect of SFRP2 and SFRP3 on hair follicle growth

**Figure 4:**
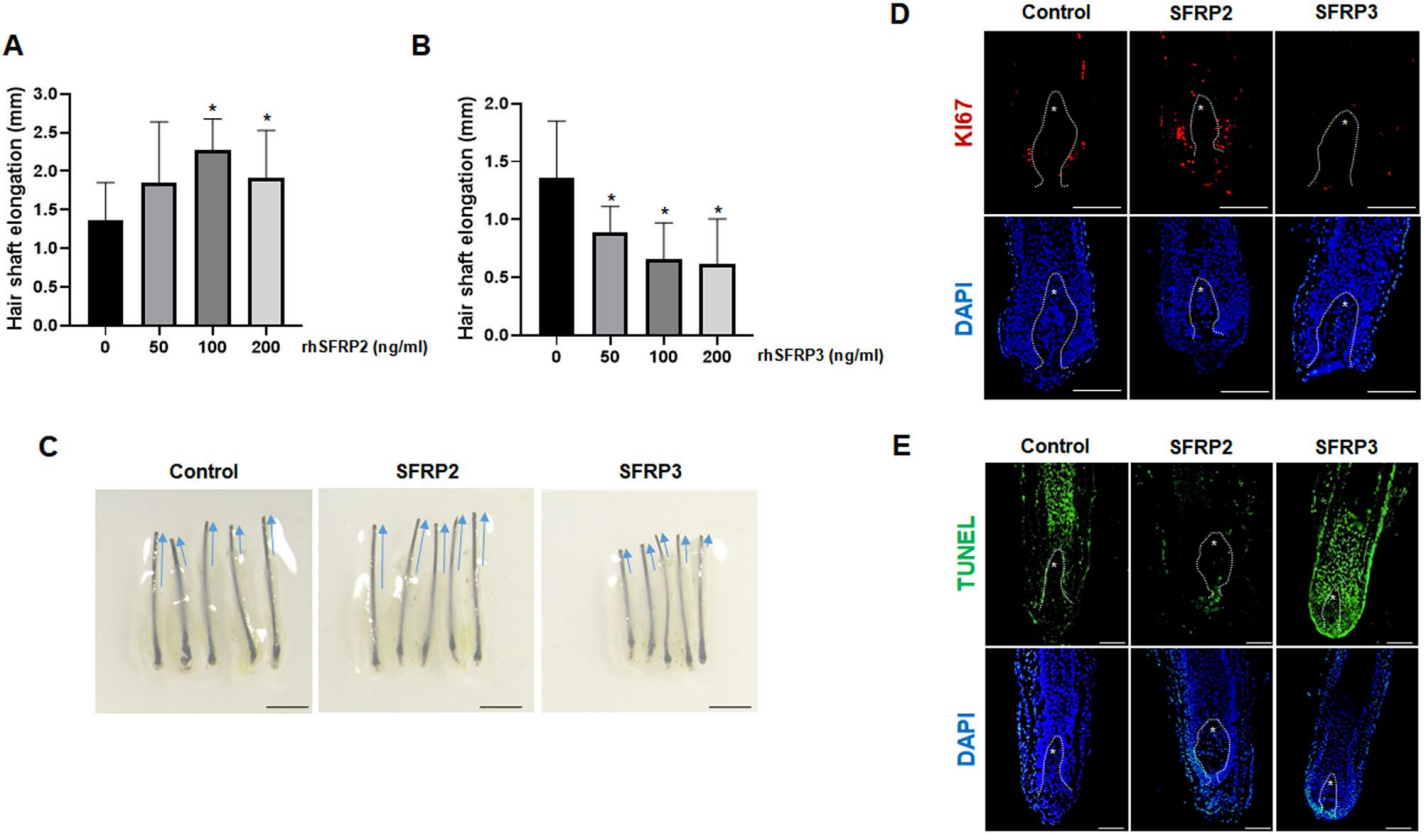
The effect of SFRP2 and SFRP3 on hair follicle growth. Human hair follicles were trimmed and incubated with increased doses of either (**A**) rhSFRP2 (50,100,200) ng/ml or (**B**) rhSFRP3 for 6 days. The length of the hair shaft was then measured compared to control (0.1% BSA). (**C**) Comparative hair shaft elongation images were also included. Bar = 1 mm. (**D**) The KI67 staining and (**E**) the TUNEL assay were also performed on the hair follicles after incubation with 100 ng/ml of rhSFRP2 or rhSFRP3 for 3 days compared to control. DAPI nuclear staining is shown in the bottom panel. Bar = 0.1 mm.

To outline the effect of SFRP2 and SFRP3 on human hair follicle, human ex vivo organ culture was performed. The hair follicles were incubated with the rhSFRP2 and rhSFRP3 in a dose dependent manner for 6 days and the hair length was measured and compared to control. The graph shows a gradual increase in the hair length of the samples treated with different concentrations of rhSFRP2 (50, 100, 200 ng/mL) (Fig. 4A,C). On the other hand, the hair length significantly decreases when treated with rhSFRP3 (Fig. 4B,C). We performed then the ki67 immunostaining (Fig. 4D) and TUNEL assay (Fig. 4E) to investigate the proliferation and apoptotic effect of SFRP2 and SFRP3 on hair follicle cells growth. Indeed, 100 ng/mL of SFRP2-treated hair follicles exhibited a retention of keratinocyte proliferation surrounding the DP, compared to the control. On the other hand, treatment with SFRP3 exhibited a lower proliferation rate by showing a lower expression of ki67-positive cells (Fig. 4D). Furthermore, hair follicles treated with SFRP2 showed a reduction in TUNEL-positive cells in the matrix and ORS layers surrounding the DP, whereas hair follicles treated with SFRP3 exhibited a significant increase in TUNEL-positive cells (Fig. 4E).

### The effect of SFRP2 and SFRP3 on ORS cells

**Figure 5:**
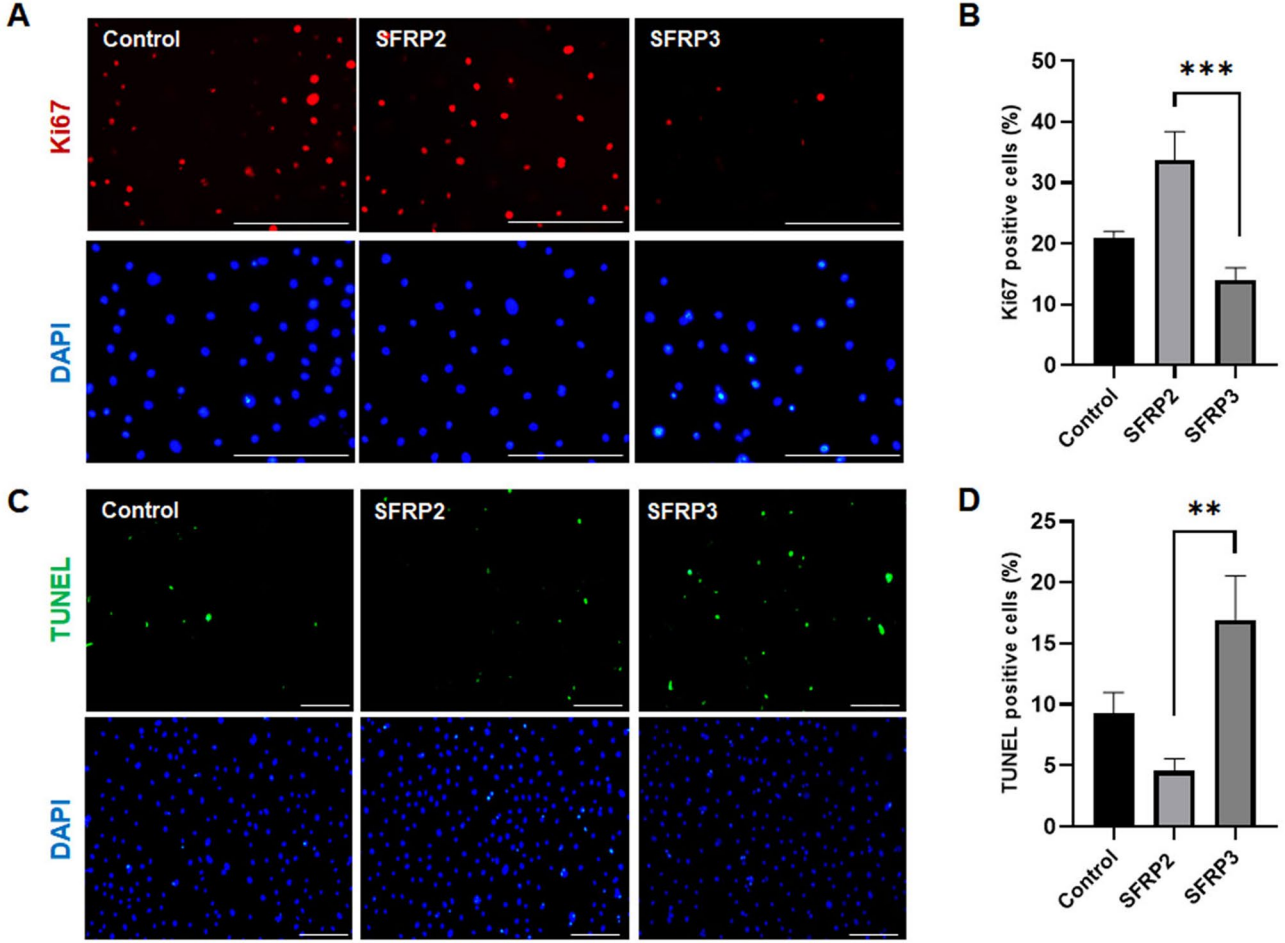
The effect of SFRP2 and SFRP3 on ORS cells. (**A**) Ki67 immunostaining was performed on ORS cells treated with 100 ng/ml of rhSFRP2 or rhSFRP3 for 24 h. DAPI was used for the nuclear staining. Bar = 0.1 mm. (**B**) Ki67-positive cells represent ORS proliferating cells among the total cell population measured and displayed in the graph using image J software. (**C**) TUNEL assay was performed to detect the apoptotic cells in ORS treated with either 100 ng/ml of rhSFRP2 or rhSFRP3 for 24 h. Bar = 0.1 mm. (**D**) Results normalization using image J software showed a significant increase in TUNEL-positive cells in the presence of rhSFRP3 compared to rhSFRP2 treatment.

Next, to understand the impact of SFRP2 and SFRP3 secreted from DP on the surrounding ORS keratinocytes, we cultured ORS cells and subsequently treated them with SFRP2 and SFRP3. ORS cells were cultured in the presence of 100 ng/mL of rhSFRP2 or rhSFRP3 for 24 h and then immunostained with ki67 and TUNEL assy. Consistent with organ culture of hair shafts, ki67-positive cells increased after treatment with rhSFRP2 compared to the control group, whereas the number of proliferating cells drastically decreased after treatment with rhSFRP3 (Fig. 5A,B). Unlike ki67 immunostaining, treating ORS keratinocytes with SFRP3 resulted in an increase in TUNEL-positive cells (Fig. 5C,D).

## Discussion

The capability of the human mini-organ hair follicle to synthesize and metabolize stress-induced neurohormones has been addressed in different studies^4,12^ which have demonstrated its characteristic role in the peripheral hypothalamic–pituitary–adrenal (HPA) axis^6^. The dermal papilla cells (DP cells), specialized mesenchymal cells, represent a crucial compartment of the hair follicle cells and display an important role in the growth and morphogenesis of the hair follicle. Thus, DP cells are considered as a major target to understand the cells response to different internal and external stimuli and their interaction with the surrounding cells mediated by their paracrine effect^13^. In this study, DEX was integrated to investigate the specific responses of the human hair follicle cells to the stressing agent. Because of its equivalence to the glucocorticoid, DEX has been the center of various studies for its versatile use as an anti-inflammatory and immunosuppressant effect. DEX has also been the focus of articles addressing the stress effect on human hair follicles^12,15^ by causing the inhibition of the proliferation of human DP cells and ORS cells. DEX has also induced the expression of DKK1, a known catagen promoter due to the blocking of Wnt/β-catenin signaling^16^ and irradicated the viability of ORS keratinocytes through apoptosis by noting the increase of the pro-apoptotic protein Bax, an effect mediated by the mesenchymal-epithelial crosstalk^17^. Belonging to a different class but serve the same purpose as DKK1, SFRP2 and SFRP3, two of five SFRP family members, are glycoproteins in charge of the regulation of the activity of Wnt signaling.

**Figure 6:**
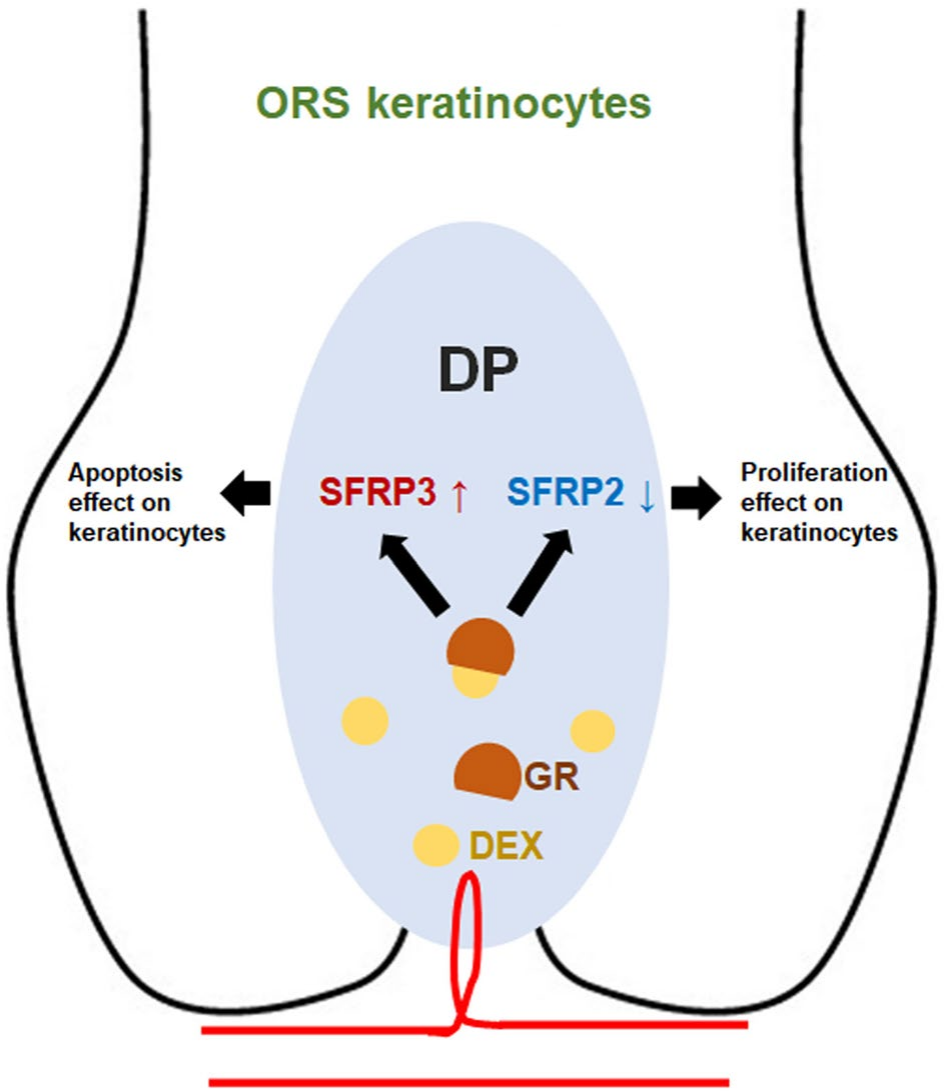
Illustration summarizing the DEX role in mediating the expression of SFRP2 and SFRP3 in DP and their respective effect on the proliferation of ORS cells in human hair follicle. DEX reduced the expression of SFRP2 and induced the expression of SFRP3 in DP cells. SFRP2 exerted a proliferating effect while SFRP3 induced apoptosis in ORS cells resulting in the regression of the hair shafts elongation.

The SFRP2 and SFRP3 proteins possess structural similarity to CRD (cysteine-rich-domain) of frizzled receptors allowing their binding to Wnt ligands. Forming the Wnt-SFRP complex, the SFRP members prevent the interaction of Wnt ligands with their corresponding frizzled receptors in a competitive matter modulating the transduction of their signaling and as a result affecting the hair growth^18^. Because of their low expression in tumor, SFRP members were considered as tumor suppressors^19^. However, SFRP2 and SFRP3 activity isn’t only limited to the inhibiting effect but can also be stimulatory of the Wnt signaling pathway. Initially considered as Wnt inhibitor, the effect of SFRP2 has been demonstrated as activating in specific contexts and for certain type of cells^20–22^. On the contrary, the frizzled related protein SFRP3 interferes with Wnt signaling by binding with Wnt ligands^23^. In the endothelial cells, it has been identified as a specific biomarker of aging in mice causing a remarkable senescence of these cells and resulting in other cardiovascular complications^24,25^. It has also been addressed in another study as an inhibitor of the proliferation and morphogenesis of fibroblasts^26^. In this study, we proved the DEX regulating effect on the gene expression of SFRP family members in which SFRP2 gene expression was downregulated in comparison with the upregulated SFRP3 expression (Fig. 6). The Affymetrix chip results were confirmed by the quantification of the mRNA gene expression of SFRP2 and SFRP3 with the real time PCR by displaying an opposite expression in time and dose-dependent treatments. Their protein expression has also been confirmed with immunoblotting. The RU486, synthetic GR antagonist, blocked the DEX-reduced mRNA expression of SFRP2 and the DEX-induced expression of SFRP3 stating the GR regulatory effect on the expression of these genes in treated DP cells. Their opposite protein expressions were detected via immunofluorescence highlighting the significant decrease of the initially present SFRP2 compared to the low expression of SFRP3 stimulated by DEX in the cytoplasm of DP cells. DEX regulates many genes in DP cells. When observing genes involved in hair follicle formation, such as versican, alkaline phosphatase, and LEF-1, it was observed that gene expression was inhibited by DEX but not significantly reversed by RU486. Not all genes inhibited by DEX were reversed by RU486 (data not shown).

To get an insight on the mechanism of action of the Wnt antagonists and their effect on the Wnt/β-catenin signaling transduction under stress, we performed real time PCR and luciferase assay on DP treated with rhwnt3a, a wnt ligand known for its promoting effect on the synthesis of melanin in mouse hair follicle^27^, the proliferation and differentiation of human epithelial cells^28^ and the maintenance of the DP cells in an active state via the activation of β-catenin signaling pathway^29^. The mRNA expression of wnt target genes Axin2 of DP cells when co-treated with rhSFRP2 and rhwnt3a showed an increase compared to the treatment of rhwnt3a alone. Luciferase reporter assay has confirmed the same results where the wnt activity significantly increased when treated with rhSFRP2 and rhwnt3a. The β-catenin activation assay detected with immunostaining (Fig. 3C) showed similar results. Surprisingly, treatment with rhSFRP3 combined with rhwnt3a has failed in inhibiting the β-catenin activity stimulated by wnt3a. These results prove the stimulating effect of the SFRP2 while binding to the wnt ligand wnt3a resulting in the enhancing of the transduction of the wnt/ β-catenin signaling contrarily to SFRP3 that refuted its anticipated effect in blocking the wnt β-catenin signaling upon binding to wnt ligands.

For further investigation of the functional effect of SFRP2 and SFRP3 on the ORS cells. The cell proliferation assay delivered by the ki67 immunofluorescence staining has noted the significant increase of ki67- positive cells in response to the treating milieu containing the rhSFRP2. In the contrary, the number of ki67-positive cell was totally decreased after treatment with 100 ng/ml of rhSFRP3 unlike rhSFRP2. The increasing effect of rhSFRP2 and the inhibiting effect of rhSFP3 on the hair shaft elongation have been confirmed via the organ culture. The ki67 immunocytochemical staining and the TUNEL assay highlighted the proliferating effect of SFRP2 and the apoptotic effect of SFRP3 on the treated hair follicles.

Although in this study, SFRP2 effect on hair follicle cells proliferation, wnt/β-catenin activation and the hair shaft elongation were rather positive, SFRP3 fulfilled its predictable effect as a wnt antagonist by inhibiting cell proliferation but it didn`t block the wnt3a activity in activating the β-catenin signaling. In fact, and in accordance with a previously reported study, SFRP3 showed a similar effect in the osteoblast proliferation where it decreased cell number by inhibiting the cell proliferation rate rather than causing cell apoptosis along with a failed decrease in β-catenin activity in the presence of wnt3a^30^.In a recent study, the phylogenetic analyzing of the SFRP members showed that SFRP2 and SFRP3 belong to 2 different subgroups where SFRP2 bind to wnt ligands, wnt3a, extracellularly whereas SFRP3 does not. Instead, SFRP3 can modulate β-catenin activity intracellularly^31^. Further investigations in that study showed that the binding domain of SFRP2 and SFRP3 to β-catenin is different where SFRP2 binds to the N terminus of β-catenin with its N terminus while SFRP3 only (as well as SFRP4) bind to the C terminus of β-catenin with their C termini^31^. Perhaps this difference in binding domains explain the different role of SFRP2 and SFRP3 in hair follicle cells and their effect on wnt/ β-catenin signaling.

Collectively, these data conclude that DEX-induced expression of SFRP3 is responsible for inhibiting proliferation and causing apoptosis of the ORS cells that results in the decrease of human hair shaft length highlighting the SFRP3 crucial role in causing hair loss. On the other hand, DEX reduced the expression of SFRP2 in charge of the proliferation of keratinocytes and the elongation of hair shaft, a potential therapeutic target for hair growth.

## Materials and methods

### Isolation and culture of human hair follicles and dermal papilla

All the conducted experiments and the used non-balding scalp specimens were obtained with consent of androgenetic alopecia patients for research purposes respecting the principles of the declaration of Helsinki, and approved by Kyungpook National University Hospital (Daegu, Korea, IRB Number KNUH 2021-09-006).

Hair follicles obtained from the biopsies were trimmed to remove the sebaceous glands and the surrounding tissue (epidermis). The trimmed follicles were placed in 24-well plates and incubated with William’s E medium. (Sigma, St. Louis, MO, USA) in William’s E medium (Sigma, St. Louis, MO, USA) with 2 mM L-glutamin (Gibco), 10 ng/ml Hydrocortisone (Sigma), 10 μg/ml Insulin (Sigma), Penicillin/Streptomycin (Gibco) in the presence of 50, 100, and 200 ng/mL rhSFRP2 or rhSFRP3 (R&D Systems, Minneapolis, USA) for 6 days in a humidified atmosphere 5% CO_2._ The hair shaft length was measured after incubation using the IMTcanUSB3.0_ISP6.3 built-in software.

Dermal papilla (DP) cells were isolated from the bulb of dissected hair follicles and transferred into collagen type 1 plastic plates (Corning, NY, USA) in DMEM supplemented with 20% FBS (fetal bovine serum), penicillin (100 U/ml) and streptomycin (100 µg/ml). The outer root sheath (ORS) cells were isolated from hair shaft, trimmed, and immersed in 20% DMEM. 3 days later, the culturing medium was changed to EPiLife medium (Gibco BRL, California, USA) containing 1% antibiotic–antimycotic solution and 1% EpiLife defined growth supplement. Both cells were harvested with trypsin and sub-cultured at 1:3 split ration after reaching cell confluency. DP cells were maintained in 10% DMEM whereas ORS cells were incubated in EpiLife with supplement. Both of cells were used at a second passage for chemical treatments.

### Cell Counting Kit-8 (CCK-8) cell viability assay

DP cells were plated in a non-coated 96-well plate (1000 cells/well) and incubated for 24 h in 10% DMEM. DP cells were then incubated in the presence or absence of different concentrations of dexamethasone (DEX, Sigma, Louis, MI, USA) for 3 days in a free DMEM medium. CCK-8 reagent (Dojindo, Kumamoto, Japan) was added at 5μL volume to determine cell viability and cytotoxicity induced by DEX. The optical density of cells was measured at 595 nm wavelength.

### Microarray analysis

The DPs isolated from hair follicles obtained from non-balding scalp of the androgenetic alopecia patients undergoing hair transplantation procedure, were cultured and then incubated with 100 nM DEX treatment for 24 h. RNA was isolated using the RNeasy Mini Kit (Qiagen, TX, USA). The microarray was carried out once with a single set of RNA samples from a patient. Fragmented cRNAwas hybridized to the HuGene-1_0-st array (Affymetrix) at 45 °C for 16 h, according to the Affymetrix standard protocol. After hybridization, the arrays were washed in a GeneChip Fluidics Station 450 with a non-stringent wash buffer at 25 °C, followed by a stringent wash buffer at 50 °C. After washing, the arrays were stained with a streptavidin–phycoerythrin complex. After staining, the intensities were determined using a GeneChip scanner 3000 (Affymetrix), controlled by GCOS Affymetrix software.

### Immunofluorescence staining

DP cells and ORS cells were plated in 8-chamber slides (Thermo Scientific, NY, USA). Each chamber was plated by 15,000 of DP cells and starved for 24 h in serum free DMEM. ORS cells were plated 30,000 cells per chamber and then cultured in EpiLife without supplement. Hair follicles used for immunostaining were placed in cryomolds using the OCT compound (Tissue-Tek: Miles, Napierville, USA) at −80 °C. For the hair follicle samples, tissue blocks then were cut into 9 um sections using the cryostat machine (Leica CM3050S, Heidelberg, Germany) and attached to glass slides. The tissue sections were fixed with 4% paraformaldehyde (PFA) for 10 min, washed 3 times with PBS (phosphate-buffered saline) and then blocked with 5% donkey serum at room temperature for 1 h. Slides were incubated with antibodies against GR (1:100 dilution; R&D Systems, Minneapolis, CA, USA), SFRP2 (FRP-2) (1:100 dilution; Santa Cruz, Delaware, CA, USA), SFRP3 (1:100 dilution; FRZB antibody, LifeSpan BioSciences, Seattle, USA), Ki67 (Becton Dickinson, Franklin, LJ, USA) or β-catenin ( Cell signaling Technology, Danvers, Massachusetts, USA) at 4 ℃ overnight. The slides were washed three times with PBS and incubated with Alexa Fluor 488-labeled donkey anti-rabbit or anti-mouse secondary antibody (Invitrogen, Eugene, OR, USA) for 1 h at room temperature. The slides were washed 3 times with PBS and then counterstained with 4′,6- diamidino 2-phenylindole (DAPI) for 10 min.

### Immunoblotting

Total cell extraction reagents (Pierce, Rockford, IL, USA) were used according to the manufacturer’s protocol to extract proteins. The extracted proteins were separated by 10% SDS-PAGE, transferred to nitrocellulose membranes, blocked with 5% skim milk in PBST for 1 h and then probed with SFRP2 rabbit polyclonal antibody (1:500 dilution; Proteintech, USA) or with rabbit polyclonal antibody against SFRP3 (FRZB) (1:1000 dilution, LifeSpan Biosceiences, Inc). HRP-conjugated donkey anti-rabbit Ig (Jackson Immuno-Research, Baltimore, PA, USA) was used as secondary antibody at 1:7000 dilution. ECL Plus was used for bands visualization (Amersham, Buckinghamshire, UK) and mouse monoclonal antibody against actin (1:5000; Chemichon, Temecula, CA, USA) was used.

### TUNEL assay

The ApopTag Fluorescein In Situ Apoptosis detection kit (EMD Millipore, Billerica, MA, USA) has been used to detect the cells undergoing apoptosis. The cryosectioned tissues of the hair follicles were fixated with 1% PFA for 10 min, post fixated with ETOH: acetic acid in a in a 2:1 ratio for 5 min in −20 ℃ and then incubated in working strength TdT enzyme mixed with the reaction buffer for 1 h in 37 ℃, then followed by the incubation in the presence of anti-digoxigenin conjugate.

### Real-time PCR analysis and RT-PCR

Total RNA was extracted by using RNeasy mini kit (Qiagen, Hiden, Germany). The RNA quantification and purification value were measured using the Nanodrop technology (Wilmington, DE, USA). 3 μg of the total RNA was used to synthesize cDNA using the ImProm-II™ kit of the reverse transcriptase kit and the oligo-dT primer in correspondence to the manufacturer’s protocol (Promega, Wisconsin, USA). For real-time PCR, the amplification of cDNA for quantification of mRNA expression of target genes was performed by adding 1 µL of DNA samples into a mixture of SYBR green (Luna Universal qPCR master mix, New England) and RNase-free water (DEPC-treated water) in a total volume of 20 µL/well in a 96-well plate for 40 cycles using a real-time PCR machine. Data were analyzed according to the manufacturer’s instructions. The data analyze was performed according to the manufacturer instructions of the real-time PCR.

For RT-PCR, the GoTaq Flexi DNA polymerase kit was supplied with 5X Green GoTaq Flexi buffer, magnesium chloride (MgCl2) solution, dNTP (2.5 mM), and nuclease-free water. A specific primer for the targeted gene was added to the mixture to amplify 1 µL of cDNA. The required amplification temperature was dependent on the primers used. The primers used for qPCR were designed and verified using nucleotide BLAST (NCBI). The primers used were as follows: GAPDH, 5′-GCCAAGGTCATCCATACAAC-3′ (forward sequence) and 5′GTCCACCACCCTGTTGCTGTA-3′ (reverse primer); SFRP2 (HS-SFRP2-1), the Qiagen product was used for real time PCR and 5′-TTCTCCTACAAGCGCAGCAA-3′ (forward) and 5′-TGGCTGGATGGTCTCTA-3′ (reverse) primers were used for RT-PCR; SFRP3, 5′-CCCCGATCTGCTCTTCC-3′ (forward) and 5′-ACTTACAGGGCTTGATGGGC-3′ (reverse) for real time PCR and 5′-TTCCACGGGACACTGTCAAC-3′ (forward) and 5′- TTCCACGGGACACTGTCAAC-3′ (reverse) for RT-PCR.

### Reporter assay

The luciferase assay was performed following the protocol of the reporter kit where DP and ORS cells (10^5^ cells per well) were transfected with pTopflash plasmid 450 ng that carries the TCF enhancer- binding promotor (Addgene, Cambridge, MA, USA) followed by the luciferase gene and 50 ng of the renilla luciferase vector used as internal control (Promega, Madison, WI, USA). After 24 h, the cells were treated with 250 ng of rhwnt3a and 100 ng of rhSFRP2 and rhSFRP3 each for 12 h.

### Statistical analysis

Data are expressed as means ± standard deviation (SD). ANOVA was used for statistical analysis of the data. P < 0.05 was considered statistically significant.

### Ethical approval

The study was conducted according to the Declaration of Helsinki Principles. Informed written consent was obtained from the patient. The Medical Ethical Committee of the Kyungpook National University Hospital (Daegu, Korea) approved all of the described studies (IRB Number KNUH 2021-09-006).

### Supplementary Information

Supplementary Information.

## Data Availability

The data sets used and/or analyzed during the current study are available from the corresponding author on reasonable request.
