# Supplementary material for: The role of dexamethasone in mediating the contradictory effects of Wnt antagonists SFRP2 and SFRP3 on human hair follicle growth
Source: Sci Rep. 2023 Oct 2;13:16504. doi: 10.1038/s41598-023-43688-5 (PMC10545675; doi:10.1038/s41598-023-43688-5)
Supplement: Supplementary file 1 — Supplementary Information. [file 41598_2023_43688_MOESM1_ESM.pptx]

## Slide 1
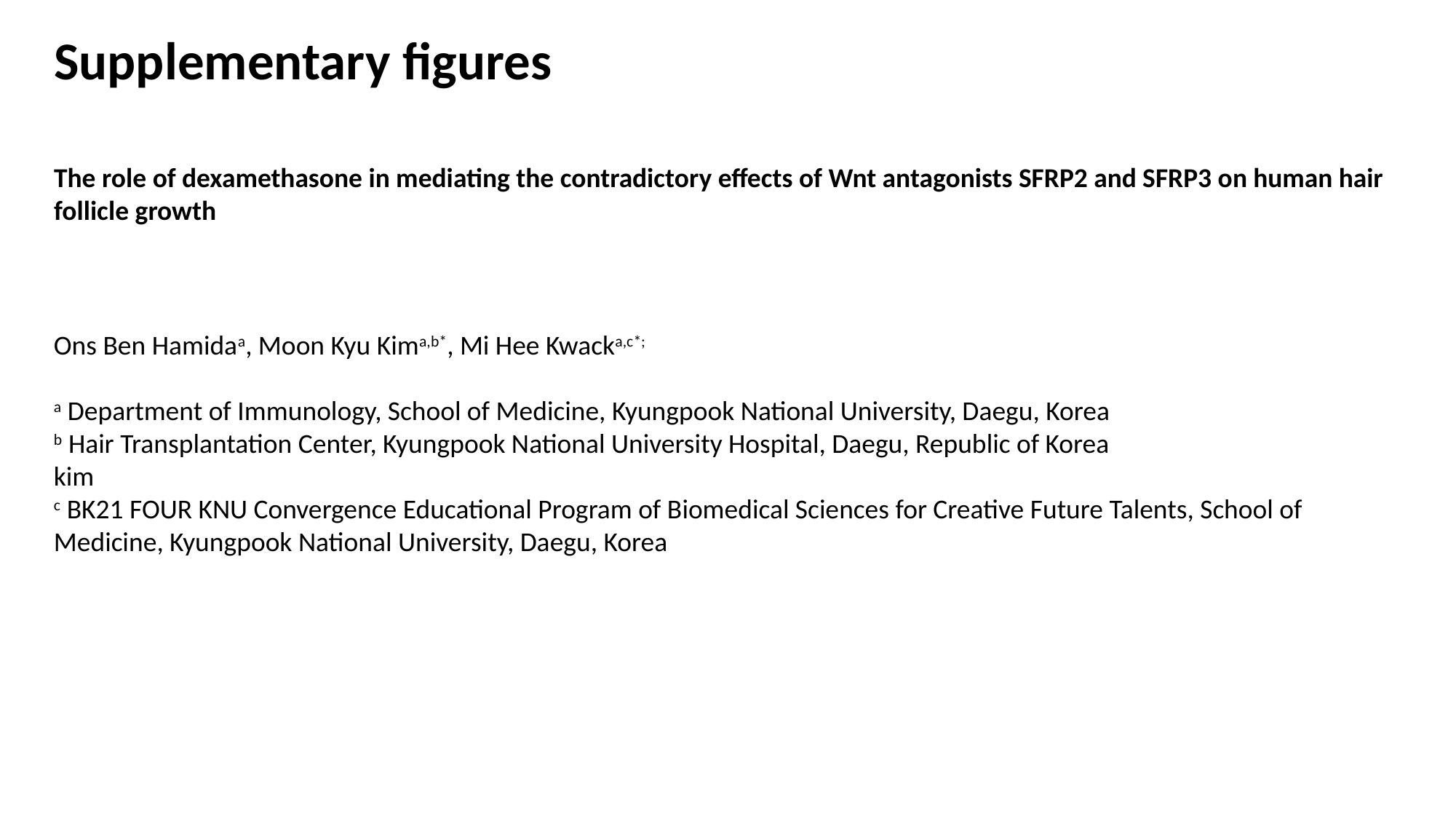

Supplementary figures
The role of dexamethasone in mediating the contradictory effects of Wnt antagonists SFRP2 and SFRP3 on human hair follicle growth
Ons Ben Hamidaa, Moon Kyu Kima,b*, Mi Hee Kwacka,c*;
a Department of Immunology, School of Medicine, Kyungpook National University, Daegu, Korea
b Hair Transplantation Center, Kyungpook National University Hospital, Daegu, Republic of Korea
kim
c BK21 FOUR KNU Convergence Educational Program of Biomedical Sciences for Creative Future Talents, School of Medicine, Kyungpook National University, Daegu, Korea

## Slide 2
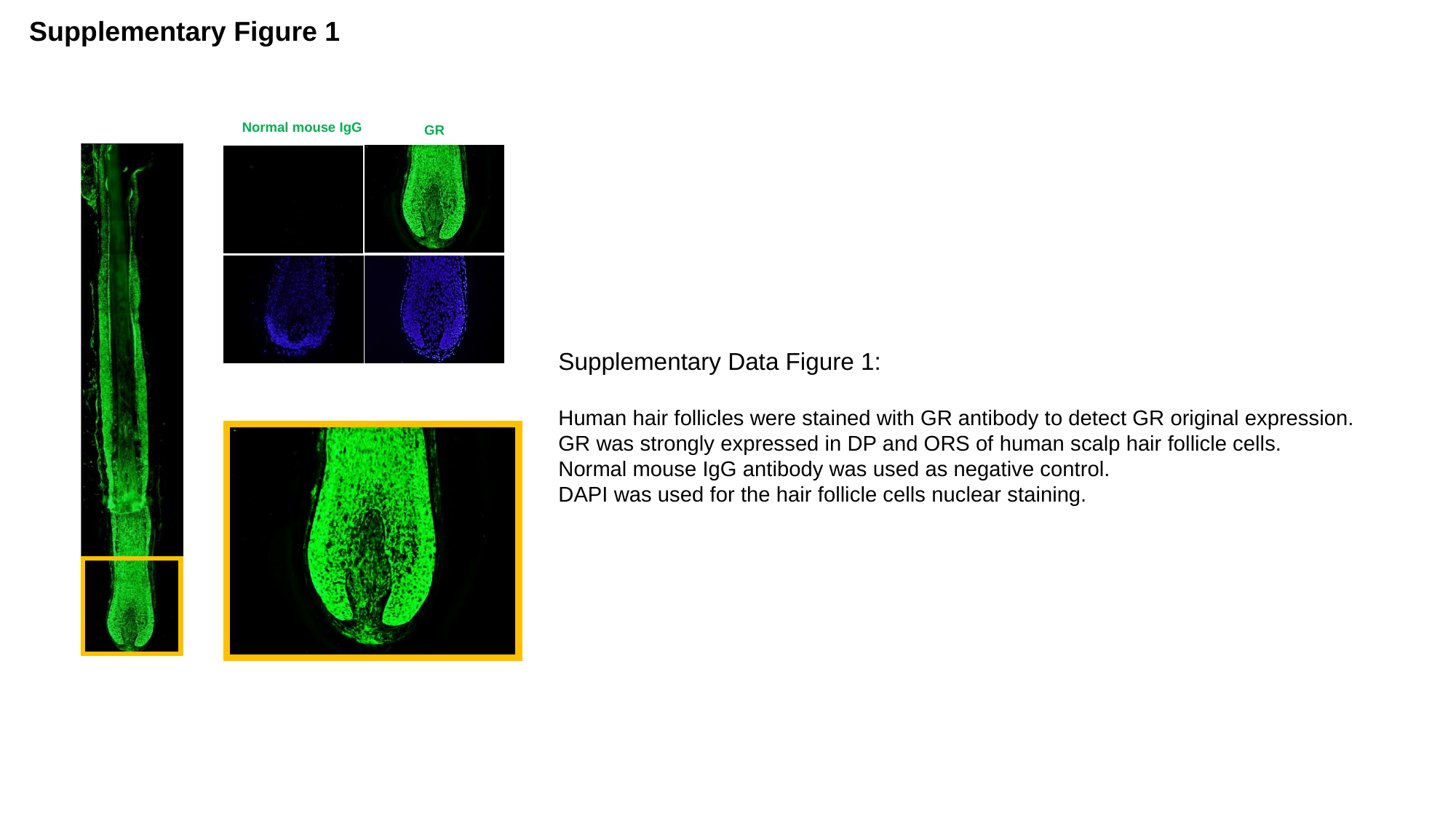

Supplementary Figure 1
Normal mouse IgG
GR
Supplementary Data Figure 1:
Human hair follicles were stained with GR antibody to detect GR original expression.
GR was strongly expressed in DP and ORS of human scalp hair follicle cells.
Normal mouse IgG antibody was used as negative control.
DAPI was used for the hair follicle cells nuclear staining.

## Slide 3
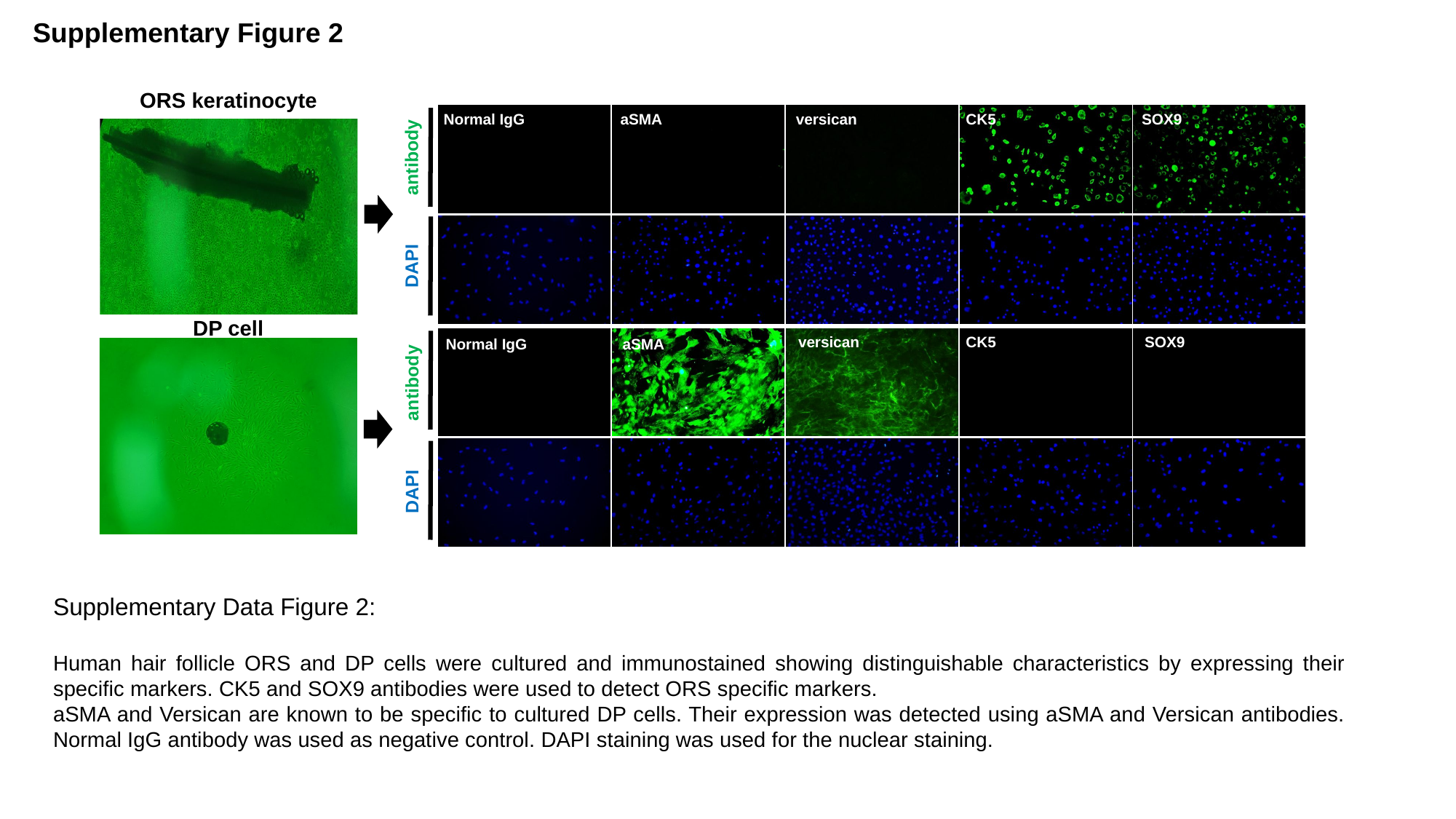

Supplementary Figure 2
ORS keratinocyte
CK5
SOX9
Normal IgG
aSMA
versican
antibody
DAPI
DP cell
versican
CK5
SOX9
Normal IgG
aSMA
antibody
DAPI
Supplementary Data Figure 2:
Human hair follicle ORS and DP cells were cultured and immunostained showing distinguishable characteristics by expressing their specific markers. CK5 and SOX9 antibodies were used to detect ORS specific markers.
aSMA and Versican are known to be specific to cultured DP cells. Their expression was detected using aSMA and Versican antibodies. Normal IgG antibody was used as negative control. DAPI staining was used for the nuclear staining.

## Slide 4
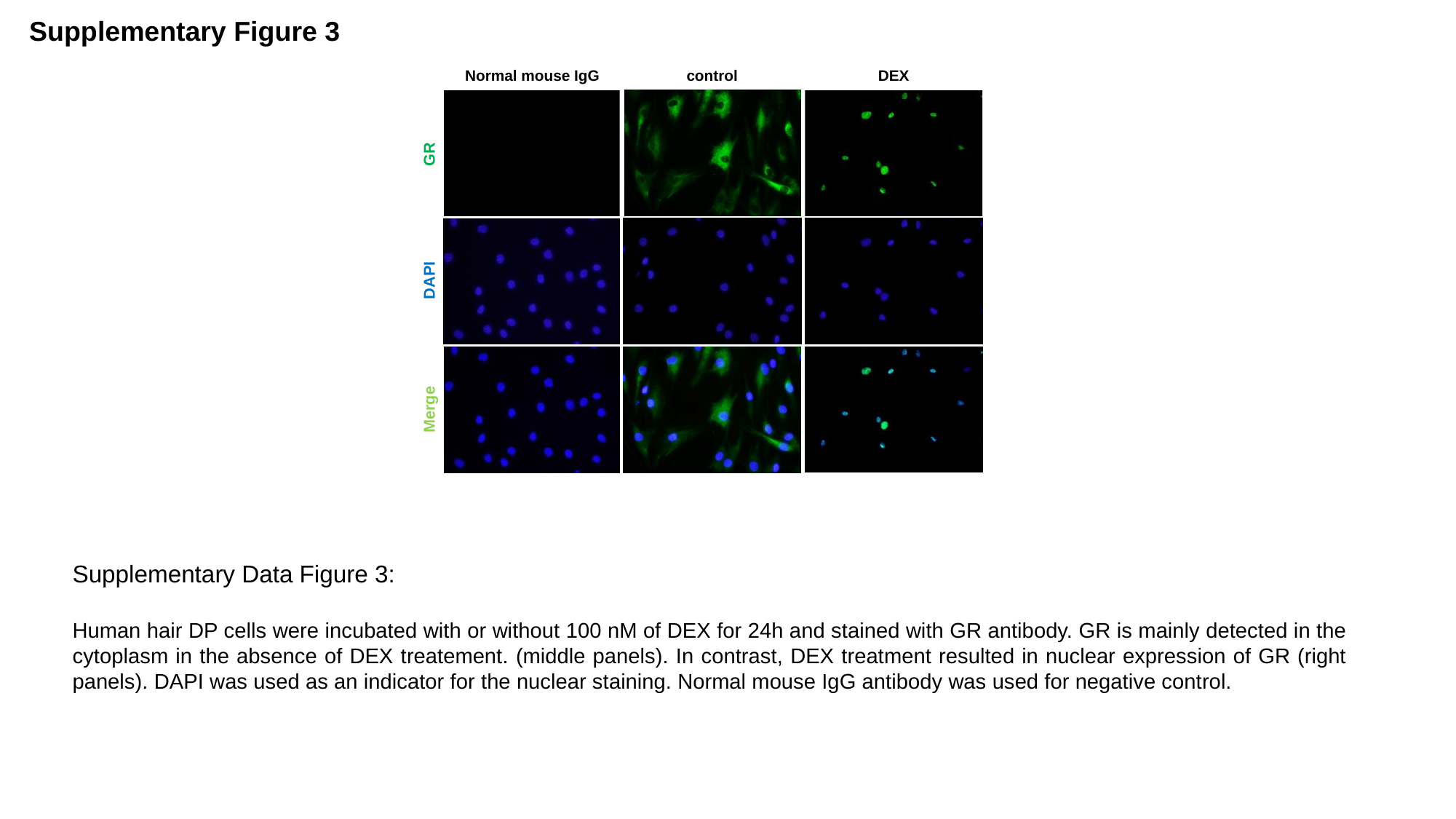

Supplementary Figure 3
Normal mouse IgG
control
DEX
GR
DAPI
Merge
Supplementary Data Figure 3:
Human hair DP cells were incubated with or without 100 nM of DEX for 24h and stained with GR antibody. GR is mainly detected in the cytoplasm in the absence of DEX treatement. (middle panels). In contrast, DEX treatment resulted in nuclear expression of GR (right panels). DAPI was used as an indicator for the nuclear staining. Normal mouse IgG antibody was used for negative control.

## Slide 5
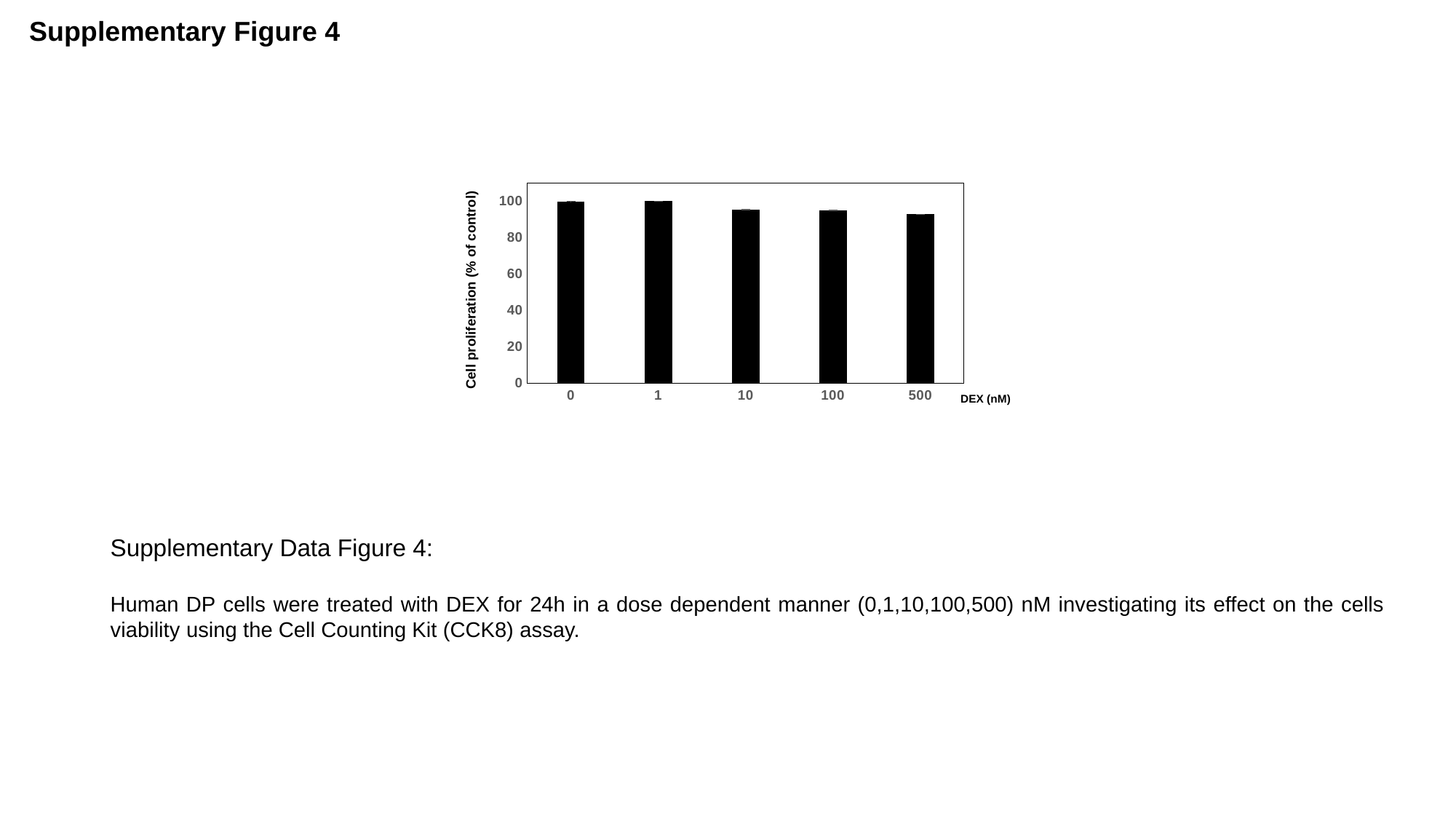

Supplementary Figure 4
### Chart
| Category | average |
|---|---|
| 0 | 100.0 |
| 1 | 100.0834424825434 |
| 10 | 95.3517235030389 |
| 100 | 95.07438451626649 |
| 500 | 92.88330528532379 |Cell proliferation (% of control)
DEX (nM)
Supplementary Data Figure 4:
Human DP cells were treated with DEX for 24h in a dose dependent manner (0,1,10,100,500) nM investigating its effect on the cells viability using the Cell Counting Kit (CCK8) assay.

## Slide 6
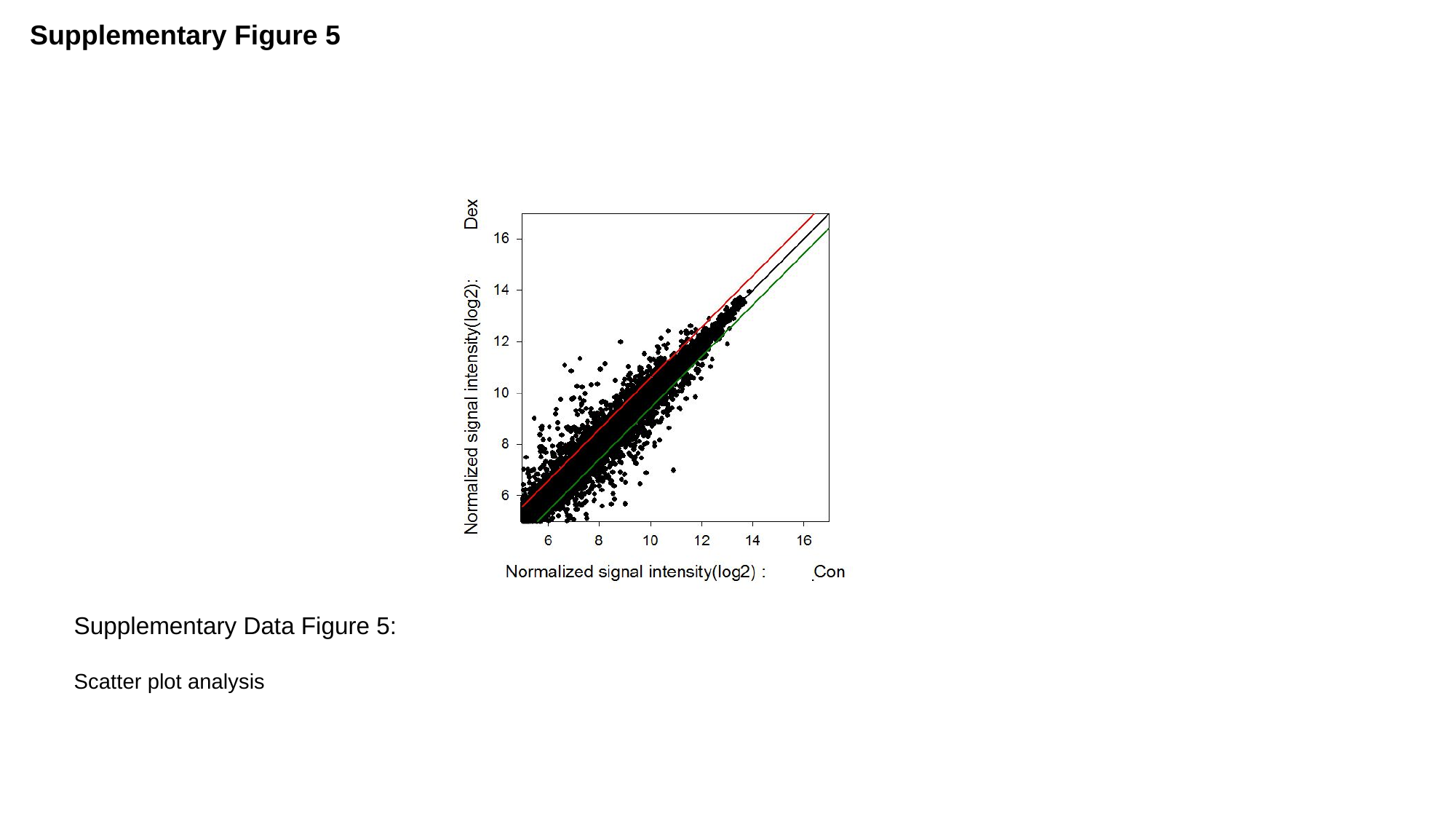

Supplementary Figure 5
Supplementary Data Figure 5:
Scatter plot analysis

## Slide 7
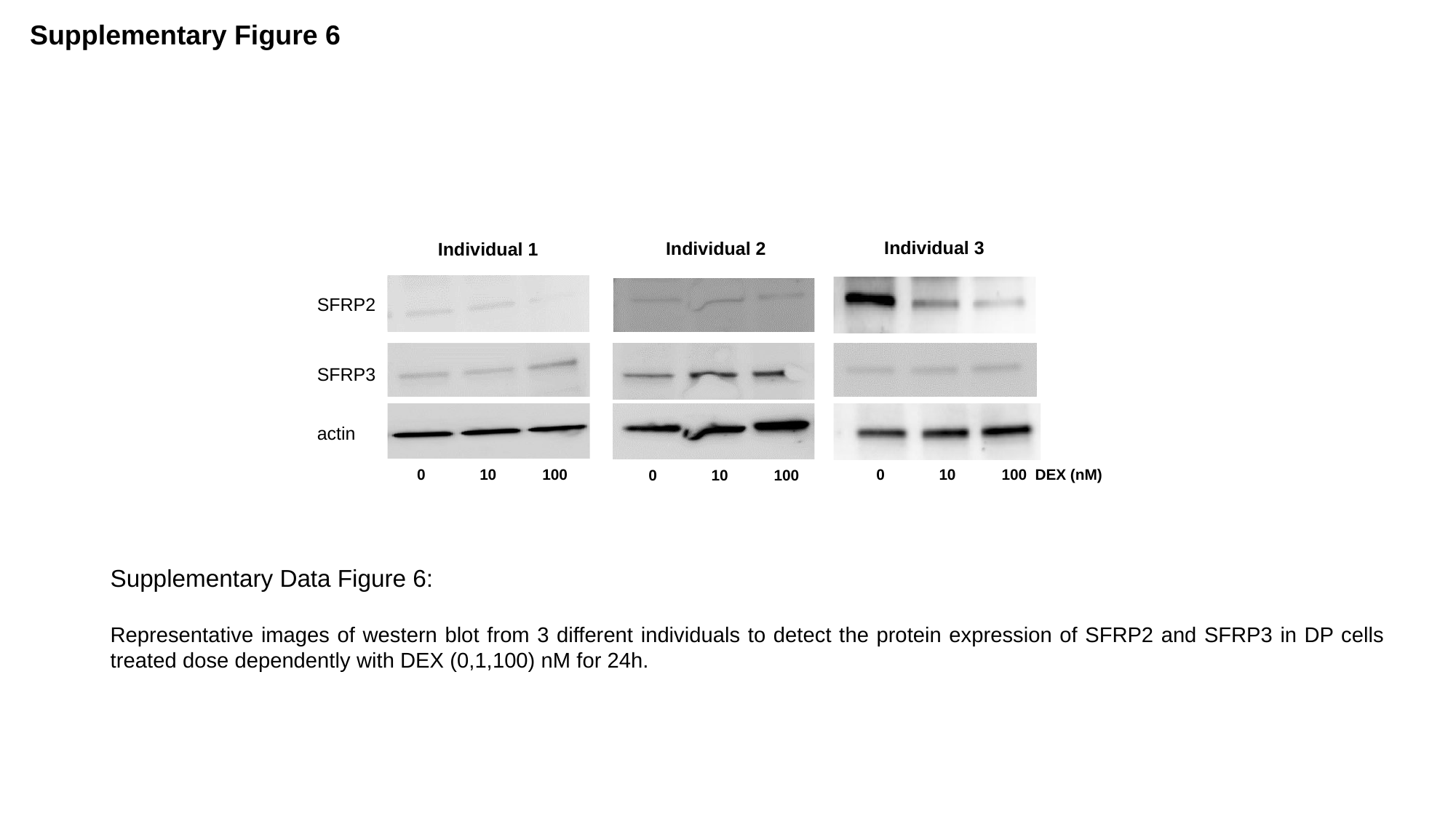

Supplementary Figure 6
Individual 3
Individual 2
Individual 1
SFRP2
SFRP3
actin
 0 10 100 DEX (nM)
 0 10 100
 0 10 100
Supplementary Data Figure 6:
Representative images of western blot from 3 different individuals to detect the protein expression of SFRP2 and SFRP3 in DP cells treated dose dependently with DEX (0,1,100) nM for 24h.

## Slide 8
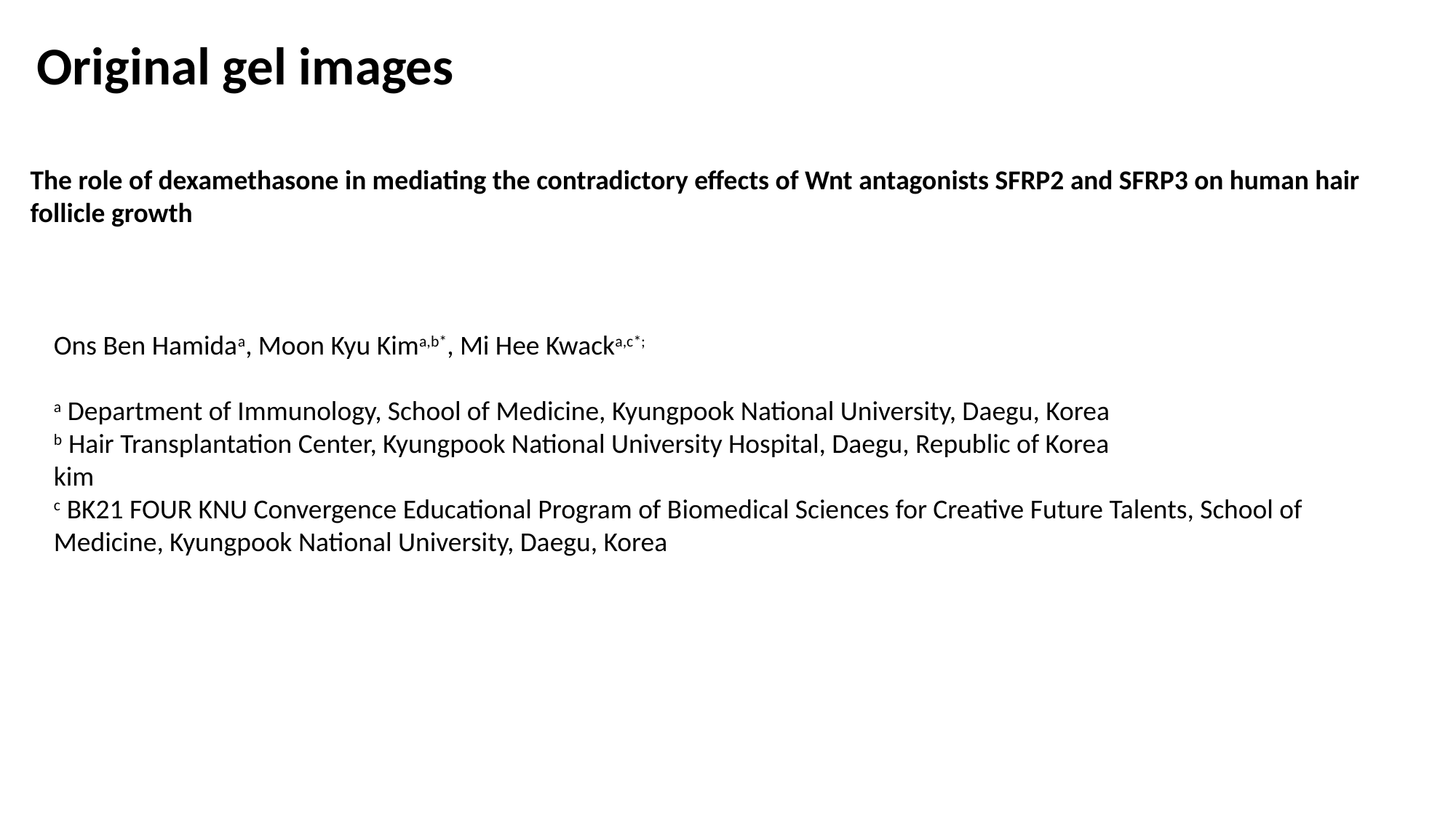

Original gel images
The role of dexamethasone in mediating the contradictory effects of Wnt antagonists SFRP2 and SFRP3 on human hair follicle growth
Ons Ben Hamidaa, Moon Kyu Kima,b*, Mi Hee Kwacka,c*;
a Department of Immunology, School of Medicine, Kyungpook National University, Daegu, Korea
b Hair Transplantation Center, Kyungpook National University Hospital, Daegu, Republic of Korea
kim
c BK21 FOUR KNU Convergence Educational Program of Biomedical Sciences for Creative Future Talents, School of Medicine, Kyungpook National University, Daegu, Korea

## Slide 9
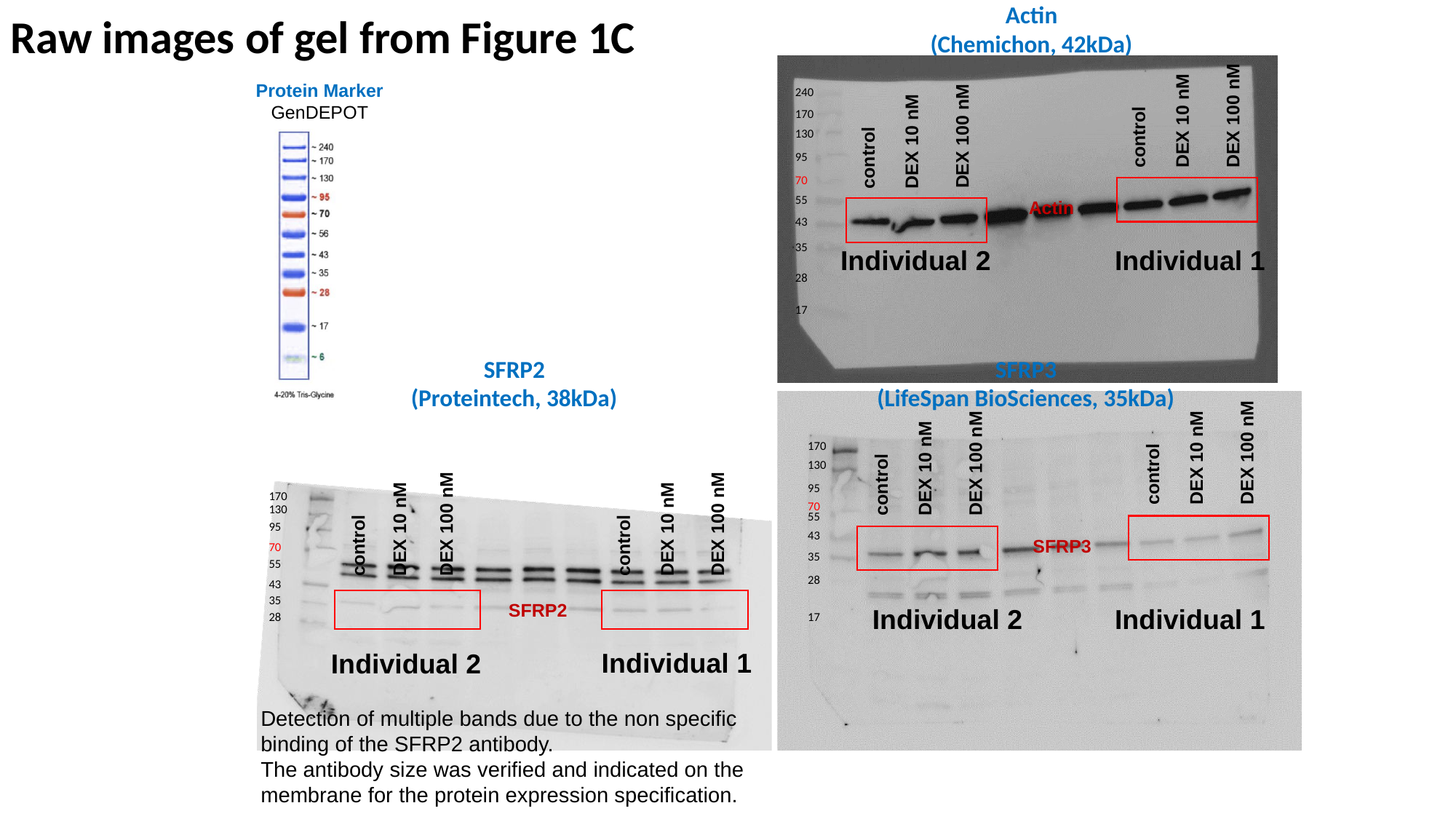

Actin
(Chemichon, 42kDa)
Raw images of gel from Figure 1C
DEX 100 nM
DEX 100 nM
control
DEX 10 nM
Protein Marker
GenDEPOT
240
control
DEX 10 nM
170
130
95
70
55
Actin
43
35
Individual 2
Individual 1
28
17
SFRP2
(Proteintech, 38kDa)
SFRP3
(LifeSpan BioSciences, 35kDa)
DEX 100 nM
DEX 100 nM
control
DEX 10 nM
control
DEX 10 nM
170
DEX 100 nM
DEX 100 nM
130
control
DEX 10 nM
control
DEX 10 nM
95
170
70
130
55
95
43
SFRP3
70
35
55
28
43
35
SFRP2
Individual 2
Individual 1
17
28
Individual 1
Individual 2
Detection of multiple bands due to the non specific binding of the SFRP2 antibody.
The antibody size was verified and indicated on the membrane for the protein expression specification.

## Slide 10
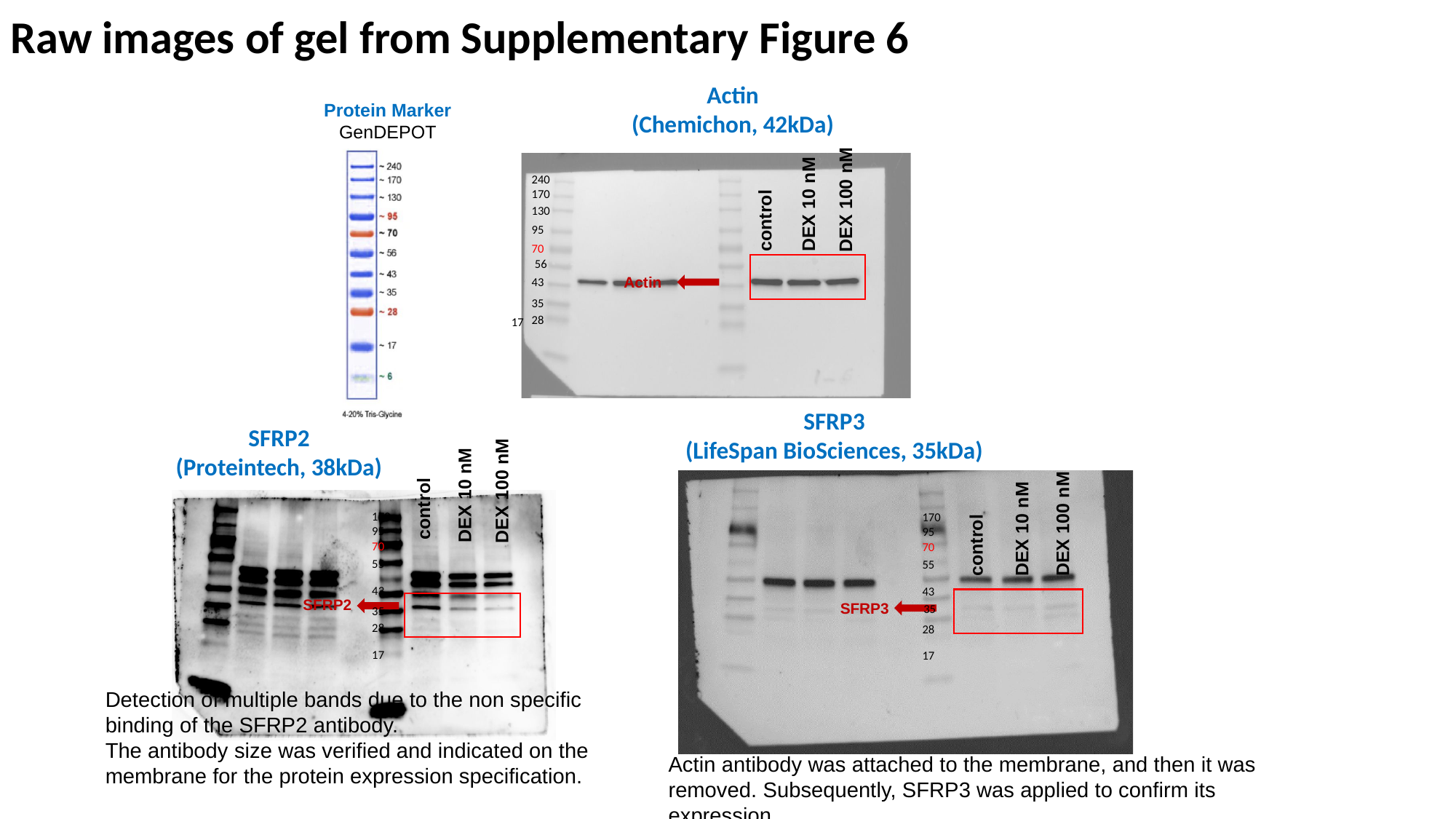

Raw images of gel from Supplementary Figure 6
Actin
(Chemichon, 42kDa)
Protein Marker
GenDEPOT
DEX 100 nM
control
DEX 10 nM
240
170
130
95
70
56
Actin
43
35
28
17
SFRP3
(LifeSpan BioSciences, 35kDa)
DEX 100 nM
SFRP2
(Proteintech, 38kDa)
control
DEX 10 nM
DEX 100 nM
control
DEX 10 nM
170
170
95
95
70
70
55
55
43
43
SFRP2
SFRP3
35
35
28
28
17
17
Detection of multiple bands due to the non specific binding of the SFRP2 antibody.
The antibody size was verified and indicated on the membrane for the protein expression specification.
Actin antibody was attached to the membrane, and then it was removed. Subsequently, SFRP3 was applied to confirm its expression.

## Slide 11
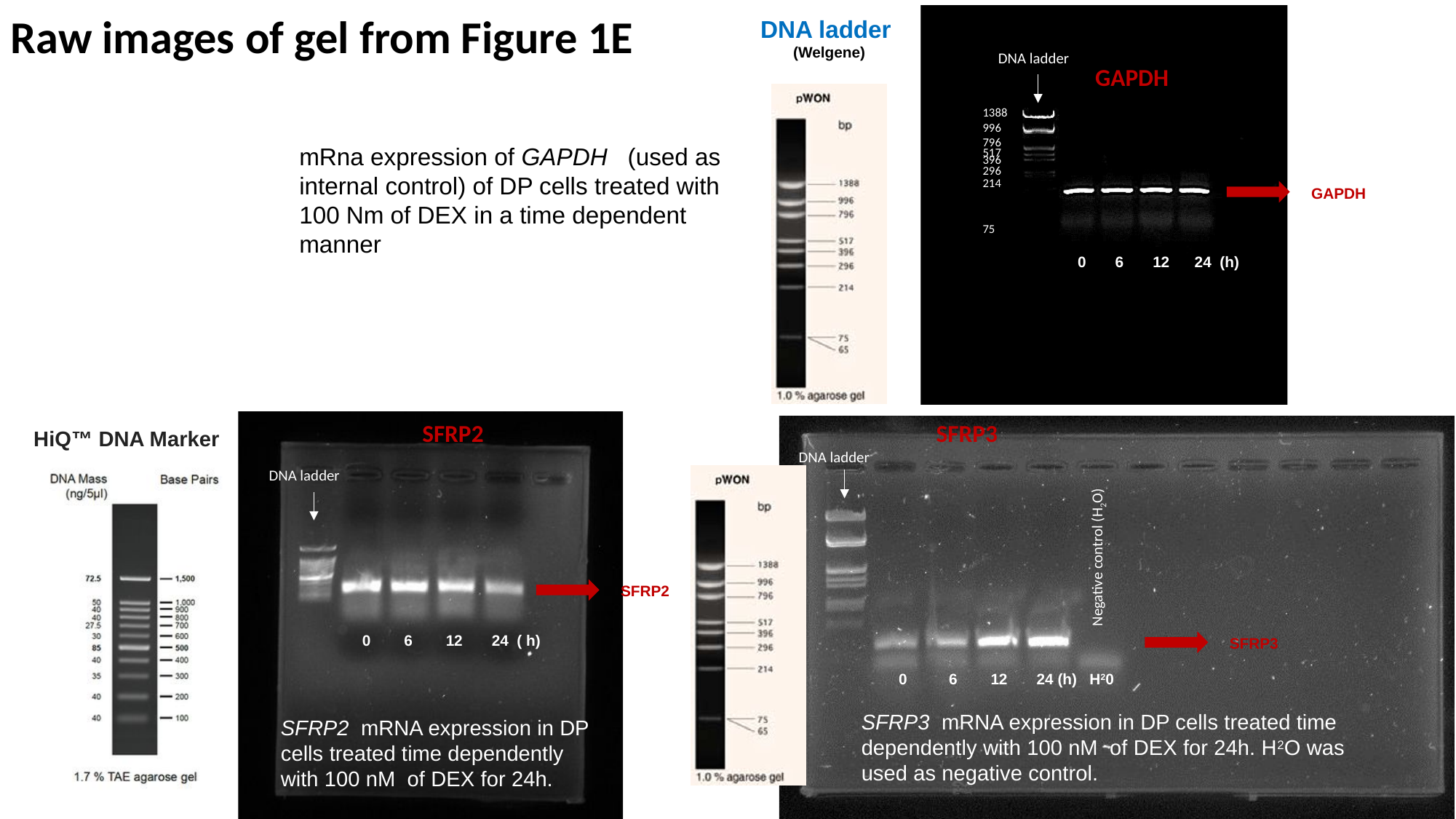

Raw images of gel from Figure 1E
DNA ladder
(Welgene)
DNA ladder
SFRP3
GAPDH
SFRP2
SFRP3
1388
996
796
mRna expression of GAPDH (used as internal control) of DP cells treated with 100 Nm of DEX in a time dependent manner
517
396
296
214
GAPDH
75
0 6 12 24 (h)
 0 6 12 24 (h)
SFRP2
SFRP3
HiQ™ DNA Marker
DNA ladder
GAPDH
DNA ladder
Negative control (H2O)
SFRP2
0 6 12 24 ( h)
 0 6 12 24 (h)
SFRP3
0 6 12 24 (h) H20
SFRP3 mRNA expression in DP cells treated time dependently with 100 nM of DEX for 24h. H2O was used as negative control.
SFRP2 mRNA expression in DP cells treated time dependently with 100 nM of DEX for 24h.

## Slide 12
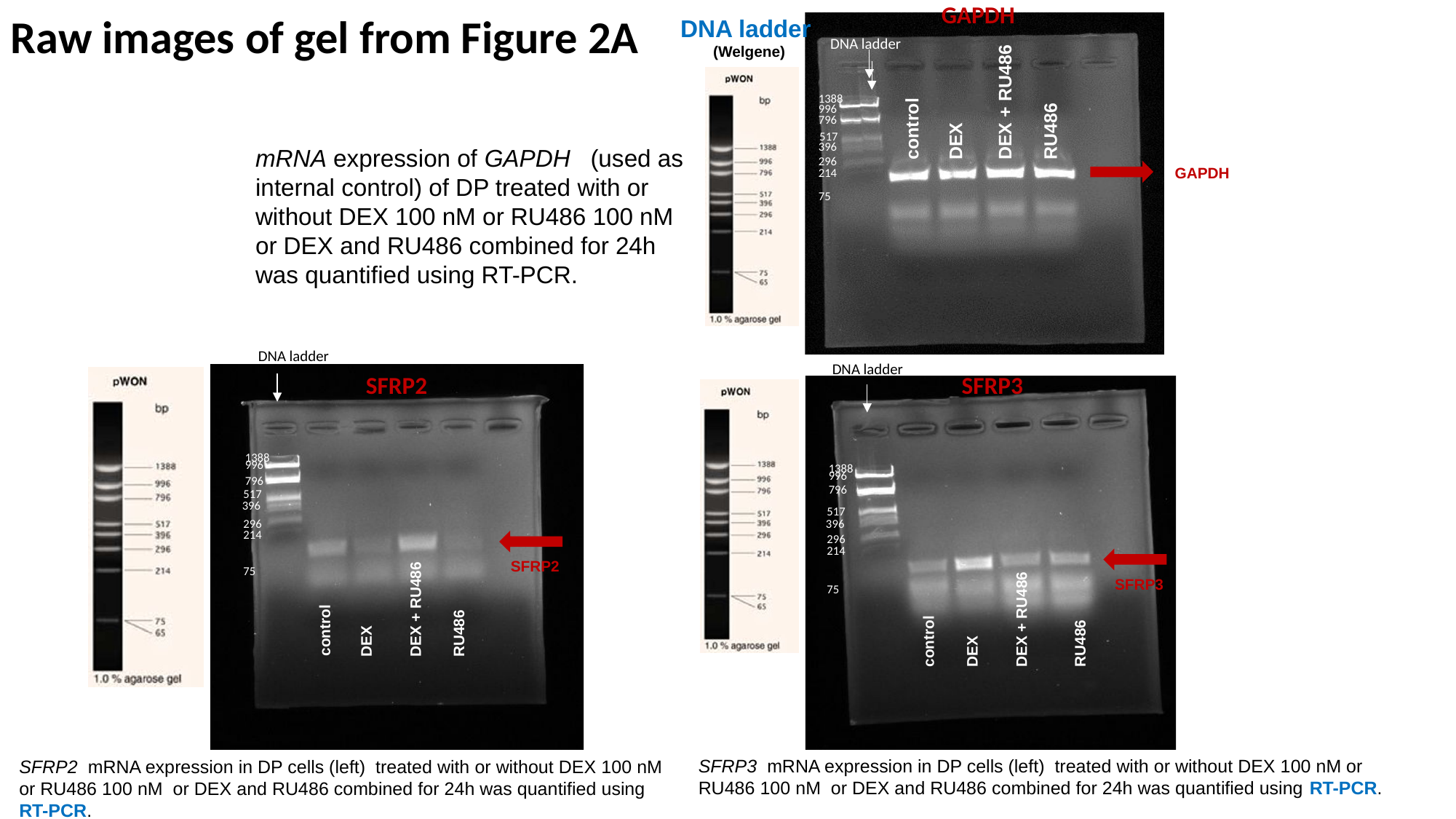

GAPDH
Raw images of gel from Figure 2A
DNA ladder
(Welgene)
DNA ladder
control
DEX
DEX + RU486
RU486
1388
996
796
517
396
mRNA expression of GAPDH (used as internal control) of DP treated with or without DEX 100 nM or RU486 100 nM or DEX and RU486 combined for 24h was quantified using RT-PCR.
296
GAPDH
214
75
DNA ladder
DNA ladder
SFRP2
SFRP3
1388
996
1388
996
796
796
517
396
517
296
396
214
296
214
SFRP2
75
control
DEX
DEX + RU486
RU486
SFRP3
75
control
DEX
DEX + RU486
RU486
SFRP3 mRNA expression in DP cells (left) treated with or without DEX 100 nM or RU486 100 nM or DEX and RU486 combined for 24h was quantified using RT-PCR.
SFRP2 mRNA expression in DP cells (left) treated with or without DEX 100 nM or RU486 100 nM or DEX and RU486 combined for 24h was quantified using RT-PCR.
